# Enlighten the People

**Published:** 1864-12

**Authors:** T. B. Welch


### ENLIGHTEN THE PEOPLE.

BY T. B. WELCH.

There is missionary work before us, Brethren. It will not do to be mere heroic Surgeons or skillful Dentists—good workmen or dignified "professors." Our calling is to *instruct* as well as relieve,—to *prevent* as well as cure. Yes, more, we must be philanthropists.

O how every right minded dentist is made to feel the truth of all this as one patient, after another calls upon him with their *unnecessary* dental troubles,—almost all brought on through ignorance or by thoughtless neglect. Instead of an inward chuckle that so many come to transfer money from their pockets to his, how can he suppress a feeling of commisseration, and an inward resolve that the masses shall be educated.

But, as in religion, many resolve, but few put into practice their resolutions; and, also, as in religion, we are too apt to do nothing, because we can not do "some great thing." "If" says one "I could write a book, would'nt I say something?"

"If I could write, says another, like a White, or a Watt, an Allport or a Leslie, *then* I would be heard in every Dental Journal." Yes, and then, perhaps, you would not be as useful as you may be now.

What every community needs, is something spoken or written directly to and for *them*, and none can do this better than an intelligent dentist living among them. Do you ask how shall this be done?

Can't you talk? You are an exception to the generality of dentists if you can not. Well than *talk*,—not altogether nonsense, as some of us are apt to do—not so incessantly with one eye upon the pocket, lest we should impart one idea without its cash equivalent,—but give them a little time —a few words a gentle hint, tending to prevent dental troubles; and not be in such haste to get at their money and see their backs.

If you can talk you can write. No matter if it is not in just the style of some great writer. *Talk it off* with your pen as though you were using your tongue, and hand it to the people in the form of a little tract, or a small pamphlet, or through some of your best newspapers; in short, weekly articles. Don't tell the people "*I*" am a great dentist,—that "*I*" have no competitor who is my equal,—that "*I*" write because no one else can,—nor "*I*"—anything else; but go before them in the simplest manner possible, with the prime object of instructing them. My word for it, if you write wisely, they will thank you and be benefited, and you shall see the fulfillment of the promise. "Cast thy bread upon the waters and after many days thou shalt find it."

